# *Mycobacterium abscessus* subsp. *massiliense mycma_0076* and *mycma_0077* Genes Code for Ferritins That Are Modulated by Iron Concentration

**DOI:** 10.3389/fmicb.2018.01072

**Published:** 2018-06-01

**Authors:** Fábio M. Oliveira, Adeliane C. Da Costa, Victor O. Procopio, Wanius Garcia, Juscemácia N. Araújo, Roosevelt A. Da Silva, Ana Paula Junqueira-Kipnis, André Kipnis

**Affiliations:** ^1^Tropical Institute of Pathology and Public Health, Department of Microbiology, Immunology, Parasitology and Pathology, Federal University of Goiás, Goiânia, Brazil; ^2^Centro de Ciências Naturais e Humanas, Federal University of ABC (UFABC), Santo André, Brazil; ^3^Collaborative Center of Biosystems, Regional Jataí, Federal University of Goiás, Goiânia, Brazil

**Keywords:** rapid growing mycobacteria, iron storage protein, pathogenic, ferritin, ferroxidase center, iron homeostasis

## Abstract

*Mycobacterium abscessus* complex has been characterized in the last decade as part of a cluster of mycobacteria that evolved from an opportunistic to true human pathogen; however, the factors responsible for pathogenicity are still undefined. It appears that the success of mycobacterial infection is intrinsically related with the capacity of the bacteria to regulate intracellular iron levels, mostly using iron storage proteins. This study evaluated two potential *M. abscessus* subsp. *massiliense* genes involved in iron storage. Unlike other opportunist or pathogenic mycobacteria studied, *M. abscessus* complex has two genes similar to ferritins from *M. tuberculosis* (Rv3841), and in *M. abscessus* subsp. *massiliense*, those genes are annotated as *mycma_0076* and *mycma_0077*. Molecular dynamic analysis of the predicted expressed proteins showed that they have a ferroxidase center. The expressions of *mycma_0076* and *mycma_0077* genes were modulated by the iron levels in both *in vitro* cultures as well as infected macrophages. Structural studies using size-exclusion chromatography, circular dichroism spectroscopy and dynamic light scattering showed that r0076 protein has a structure similar to those observed in the ferritin family. The r0076 forms oligomers in solution most likely composed of 24 subunits. Functional studies with recombinant proteins, obtained from heterologous expression of *mycma_0076* and *mycma_0077* genes in *Escherichia coli*, showed that both proteins were capable of oxidizing Fe^2+^ into Fe^3+^, demonstrating that these proteins have a functional ferroxidase center. In conclusion, two ferritins proteins were shown, for the first time, to be involved in iron storage in *M. abscessus* subsp. *massiliense* and their expressions were modulated by the iron levels.

## Introduction

The *Mycobacterium abscessus* complex, composed of *M. abscessus* subsp. *abscessus*, *M. abscessus* subsp. *massiliense*, and *M. abscessus* subsp. *bolletii* has emerged as human pathogens in the last few years (Petrini, 2006; Medjahed et al., 2010; Lee et al., 2015; Tortoli et al., 2016). Mycobacteria belonging to this complex cause several diseases in humans. These include severe lung, skin, post-traumatic, and post-surgical infections, especially in patients that have cystic fibrosis as well as in immunocompetent individuals (Medjahed et al., 2010). Due to its capacity to adapt and persist in the environment as well as to resist disinfection procedures, the infections caused by the *M. abscessus* group are usually due to cross contamination, through surgical equipment or other contaminated procedures (Cardoso et al., 2008). The transmission of *M. abscessus* has been already documented among cystic fibrosis individuals. Therefore, infections in humans can occur by both direct and indirect transmission (Bryant et al., 2013; Lee et al., 2015; Bryant et al., 2016).

Its capacity to infect and multiply within phagocytic cells indicates that *M. abscessus* can evade the defense mechanisms imposed by the host, resulting in successful infection (Martins de Sousa et al., 2010; Shang et al., 2011; Bernut et al., 2014; Abdalla et al., 2015a; Bakala et al., 2015; Caverly et al., 2015). One mechanism used by this bacillus to multiply within macrophages is the induction of Heme-Oxygenase-1 (HO-1), which reduces the toxic oxidative stress effects produced by the cell (Abdalla et al., 2015a). Part of the HO-1 action is accomplished by the reduction of free ferrous ion Fe^2+^ inside macrophages through the storage of this metal by ferritins, thus preventing the formation of free radicals by the Fenton reaction (Imlay et al., 1988; Vile and Tyrrell, 1993). Hence, mechanisms of extracellular iron level regulation are crucial for the bacilli to survive within macrophages. However, studies have shown that the intracellular levels of iron are also important for bacilli to establish infection, because both absence and excess of iron are deleterious (De Voss et al., 2000; Pandey and Rodriguez, 2012). Consequently, in order to survive within macrophages, the bacilli must be able to obtain, store, and regulate the iron levels during entire infection (Gold et al., 2001; Pandey and Rodriguez, 2014; Pandey et al., 2014).

The main protein family involved in regulating intracellular iron levels and reducing its toxic effects are the ferritins. The proteins within this family may be divided into three sub-classes: ferritin (non-heme binding), bacterioferritin (heme bound) and Dps (DNA-binding protein from starved cells) (Andrews et al., 2003). Bacterial ferritins and bacterioferritins have similar structures, and they are composed of 24 identical subunits arranged in an octahedral form, with a ferroxidase catalytic site at its center. At this catalytic site, Fe^2+^ is oxidized to Fe^3+^ and stored within its hollow interior, where it can store up to 4,500 atoms of this metal ion (Andrews, 1998; Bou-Abdallah, 2010). Consequently, iron is stored in its non-reactive form (Fe^3+^), avoiding its toxic effects on the cell, and can be released when needed (Andrews, 1998).

Despite similar structure between baterial ferritins and bacterioferritins, their amino acid sequences present low identity and bacterioferritins have a heme group suggesting different origins for these proteins (Andrews, 1998; Carrondo, 2003). *M. tuberculosis* (Mtb) has two types of ferritin-like molecules, bacterioferritin (BfrA) and ferritin (BfrB), coded by the genes Rv1876 and Rv3841, respectively (Cole et al., 1998). Crystallographic studies showed that these proteins are organized similar to the ferritin superfamily, which is an oligomer in the form of a shell with a catalytic center of ferroxidase (Gupta et al., 2009; Khare et al., 2011). Studies using Mtb mutants, which had their *bfrA* and *bfrB* genes deleted solely or together, demonstrated the importance of both in iron homeostasis, as well as in the virulence and pathogenicity of this bacillus in different infection models (Pandey and Rodriguez, 2012, 2014; Reddy et al., 2012; Khare et al., 2017).

In addition, ferritins appear to be involved in drug resistance of Mtb, because it was shown that the lack of ferritin in this bacillus increased the susceptibility to antimicrobials used to treat tuberculosis (Pandey and Rodriguez, 2012). Proteomic analysis indicated that both BfrA and BfrB were overexpressed in aminoglycosides resistant as compared to sensitive clinical isolates of Mtb (Kumar et al., 2013; Sharma et al., 2015). Additionally, overexpression of Rv3841 (*bfrB*) by recombinant *Escherichia coli* resulted in increased kanamycin and amikacin resistance (Sharma et al., 2016). Taken together, BfrA and BfrB proteins, could be promising drug targets against mycobacteria infection.

Recent studies with drugs that act in the iron metabolism of *M. abscessus* confirm that this metal is crucial for the development of this bacillus (Abdalla et al., 2015b). However, the genes and proteins involved in the iron homeostasis and their importance for establishing infection remain unclear. The present study demonstrates for the first time that *M. abscessus* subsp. *massiliense* has two ferritin (non-heme binding) proteins involved in iron storage and related in the iron homeostasis both *in vitro* and in infected macrophages.

## Materials and Methods

### *M. abscessus* subsp. *massiliense* GO06 Genome Annotation

The complete genome sequences of *M. abscessus* subsp. *massiliense* GO06 (Mycma GO06, taxid: 1198627) and the pathogen reference strain of *M. tuberculosis* H37Rv (taxid: 83332) used in this study were obtained from NCBI^1^. The BLAST tool from NCBI^2^ was used for genome and proteome annotations of the Mycma GO06 strain as well as other mycobacteria species genomes.

### Bacterial Strains and Growth Conditions

*Escherichia coli* XL1-Blue and BL21 (DE3) pLysS were used for cloning and expression of the recombinant proteins, respectively. *E. coli* strains were cultured in Luria Bertani (LB) broth (Himedia) and *M. abscessus* subsp. *massiliense* (Cardoso et al., 2008) was cultured in Mueller Hinton broth or agar at 37°C under 180 rpm shaking. For growth in different iron concentrations, the minimal medium contained 3.6 mM of KH_2_PO_4_, 2.0 mM of MgSO_4_ 7H_2_O, 6% (v/v) of glycerol, 30 mM of L-asparagine, 0.006 mM of ZnSO_4_, and 0.05% (v/v) of Tween 80, pH 6.8 in iron free conditions. For low iron conditions, media was supplemented with 1.25 mM of deferoxamine mesylate (DFO). In high iron conditions, media was supplemented with 50, 150, 300, or 450 μM of FeCl_3_. Minimal media without supplementation (iron or DFO) contains enough iron concentration to sustain *M. abscessus* subsp. *massiliense* growth, and thus this condition was used as normalizer. *E. coli* transformants were selected in medium supplemented with the antibiotics kanamycin (KAN) and chloramphenicol (CAM) at 20 μg/ml.

### Homology Models

The predicted 0076 and 0077 amino acid sequences were initially submitted to I-TASSER (Zhang, 2008) and an initial model was obtained for each sequence. I-TASSER strategy starts from the structure templates identified by LOMETS (Wu and Zhang, 2007) in the PDB library. I-TASSER only uses the templates of the highest significance in the threading alignments, which are measured by the *Z*-score. The *C*-score for each model was verified to evaluate the quality of the predictions from I-TASSER. *C*-score values are related to the expected TM-score (Zhang and Skolnick, 2004, 2005) between the model and native structure (structural similarity of two proteins).

### Molecular Dynamics Simulations

To explore the stability and conformational variability of the initial models in its native environment, molecular dynamics (MD) simulations were performed with Gromacs 5.1.3 (Berendsen et al., 1995; Yu, 2012) using force field AMBER99SB-ILDN (Yildirim et al., 2010). The proteins were solvated with a box cubic wall distance of 10 Å using water model TIP3P (Mahoney and Jorgensen, 2000). The system was neutralized by adding the required number of counter ions according of each protein. The system was initially minimized using the steepest descent energy. The simulations were complete when the tolerance no longer exceeded 1000 kJ/mol. In the next three steps consisted of 50 picoseconds MD simulations in NVT and NPT ensemble at 300 K with a restraint of 50 kcal/mol/Å on the protein atoms and 0.5 ns without restraint in NPT ensemble at 300 K. Finally, the simulations were performed for 100 ns for all proteins with a constant temperature of 300 K, 1 atm pressure, time-set of 2 fs, and without any restriction of protein conformations. All information concerning the trajectory of these times were collected every 50 ps. The equilibration of the trajectory was evaluated by monitoring the equilibration of quantities, such as the root-mean-square deviation (RMSD) (Coutsias et al., 2004) of the non-hydrogen atoms, total energy, potential energy, and kinetic energy. The conformations that best represented the structures of the entire trajectory were selected following the algorithm described by Daura et al. (1999). A cutoff of 0.2 nm for the clusters was used considering the profile of the RMSD observed for each protein. The clusters were determined using the non-hydrogen atom RMSD values. The quality of the predicted structure was assessed using the MolProbity server (Chen et al., 2010).

### Gene Expression Evaluation of *M. abscessus* subsp. *massiliense*

*Mycobacterium abscessus* subsp. *massiliense* cultures grown in minimal media with different iron concentrations were harvested by centrifugation at 3,200 × *g* for 10 min. The pellet was suspended with 1 ml of nuclease-free water (Ambio Life Technologies) and 0.25 ml of glass beads (0.1 mm in diameter, Glass Glass Disruptor Beads; USA Scientific, Inc.) was added. The suspension was maintained in ice and vortexed five times for 2 min with 30 s intervals. The Lysate was centrifuged at 12,000 × *g* for 10 min at 4°C, and the aqueous phase was transferred to a new tube for addition of 215 μl of ethanol (J.T. Baker) for each 400 μl of recovered solution. The solution was then applied to an RNA purification column (Phenol-Free Total RNA Purification, Amresco) for purification according manufacture’s instructions. The obtained RNA was treated with RNAse free DNAse (Sigma-Aldrich) and stored at -80°C until further use.

The Reverse Transcriptase M-MLV kit (Sigma-Aldrich) was used for cDNA synthesis. The reaction consisted of 200 ηg of total RNA, 0.5 mM of dNTPS and 0.63 μM of random hexamer primers (Gibco/Thermo Fisher Scientific) and was incubated for 5 min at 65°C. Next, the system was transferred to ice and reverse transcriptase buffer, 200 U of M-MLV reverse transcriptase, and 40 U of RNAse OUT (Invitrogen) were added. The reaction was incubated at 37°C for 1.5 h. Then the synthesized cDNA was stored at -20°C until its use for Real Time PCR (RT-PCR). RT-PCR was set up in a 0.2 ml tube, using 10 μl of SYBR Green mix (Bio-Rad), 0.5 μM of each primer (**Supplementary Table S2**) and 5 μl of cDNA in a final volume of 20 μl. The reaction was run on a IQ5 thermocycler (Bio-Rad). RT-PCR conditions were as follows: 95°C (5 min), 40 cycles of 95°C (15 s), 58°C (30 s), and 72°C (1 min), and at the end a melting temperature curve ranging from 70 to 99°C (ramp rate of 0.5°C per cycle and 30 s in each temperature) was performed and the detected fluorescence emission recorded. Positive samples were considered when the fluorescence surpassed the threshold baseline. The cycle of threshold crossing corresponded to the Ct value. Ct values greater than 35 were considered negative. Ct values were tabulated on an Excel 2011 spreadsheet, and the relative expression was determined with the Delta Delta Ct (2^-ΔΔCt^) method using the expression of the *16s* rRNA gene as normalizer. The calibrator condition in this study was bacteria grown in minimal media without DFO and FeCl_3_, as these conditions contain sufficient iron levels to support mycobacteria growth. The relative gene expression levels were analyzed on GraphPad Prism 7 (version Prism 7a, Graph Pad) for statistical analysis and graphic representations.

### Infection of Bone Marrow-Derived Macrophages (BMDM)

To evaluate the *ex vivo* gene expression of *M. abscessus* subsp. *massiliense*, bone marrow from C57BL/6 mice were collected (Becker et al., 2012) and submitted to differentiation as previously described da Costa et al. (2017). BMDM (1 × 10^6^/ml) were infected with *M. abscessus* subsp. *massiliense* at a MOI of 10 in a 24 well plate with or without coverslip. Three hours after infection, extracellular bacteria were removed by washing the wells twice with RPMI with 10 μg/ml of kanamycin and then adding RPMI media supplemented with 10% fetal bovine serum (FBS). The CFU determination and expression profile of the genes *mycma_0076* and *mycma_0077* during infection was assessed by recovering the bacilli at three different times: 3, 24, 48, and 72 h post infection and additionally the wells with coverslip 24 and 72 h were randomly selected to stained with Instant-Prov (NewPRQV) according to the manufactured instruction’s. At these times, the wells were randomly selected and from them the supernatant was removed and substituted with nuclease free water (Ambion) to lyse macrophages. The lysate was transferred to 1.5 ml nuclease free tubes, centrifuged at 16,000 × *g* for 10 min at 4°C and the pellet was processed for RNA extraction. The relative gene expression was determined by the delta delta Ct (2^-ΔΔCt^) method using the expression of the *16s* rRNA gene as normalizer. The bacilli, obtained from culture supernatant after 3 h of macrophage infection, was used as calibrator.

### Cloning and Expression of *mycma_0076* and *mycma_0077* Genes

The *mycma_0076* and *mycma_0077* genes were amplified by PCR using *M. abscessus* subsp. *massiliense* GO06 (Raiol et al., 2012) genomic DNA as template. The primers were designed using NCBI Primer designing tool^3^. The following primers were used to amplify the *mycma_0076* gene include: forward 5′ CATATGACCGCGACCGACACCCCGA 3′ that incorporates an *Nde*I restriction site (underlined) and reverse 5′ GGATCCTCTTGTTGACGTGCTTAGAGCG 3′ that incorporates a *Bam*HI restriction site (underlined). Similarly, the primers for the *mycma_0077* gene amplification were: forward 5′ CATATGGTGGCTACCACCGATCTCCATG 3′ and reverse 5′ CTCGAGCGCAAAATTATCAGAGCGCGC 3′ that incorporate an *Nde*I and an *Xho*I restriction sites, respectively. The PCR products of each gene were cloned into pET28a (Novagen) vector using their respective flanking sites. Recombinant plasmids were confirmed by sequencing. Recombinant protein expression was performed by transforming the recombinant plasmids into *E. coli* BL21 (DE3) pLysS cells.

### Recombinant Protein Purification

*Escherichia coli* BL21 (DE3) pLysS containing the recombinant plasmids were grown in LB containing kanamycin (20 μg/ml) and chloramphenicol (20 μg/ml) until OD_550__nm_ reached 0.5. Then the culture was induced with 1 mM of isopropryl-1-thio-β-D-galactopyranoside (IPTG) at 37°C, 180 rpm for 4 h. Cells were then harvested by centrifugation at 4,000 × *g* for 20 min at 4°C. The pellet was used for protein extraction using the commercial protein purification QIAexpress-Ni_NTA Fast Start kit (Qiagen) according to the manufacturer’s instructions. Proteins eluted from the nickel column were further purified on a gel filtration Superdex 200 10/300 GL chromatographic column (GE Healthcare). The column was previously equilibrated with 50 mM NaH_2_PO_4_ buffer adjusted at pH 8.0 containing 300 mM NaCl (pH 8.0) buffer. The column was calibrated with molecular weight standards (GE Healthcare), and chromatography was performed at a 1 ml/min rate with 5 MPa pressure and detection at 280 nm on an AKTA purifier system (GE Healthcare). Eight microliters from each collected fraction were analyzed on 12% SDS-PAGE. Protein concentration was determined by using Bradford’s reagent with bovine serum albumin as the standard.

### Mouse Anti-r0076 Antibody Production

Three C57BL/6 mice were immunized by the subcutaneous route with purified r0076 protein. In the first immunization, a formulation consisting of 50 μg of r0076 protein and 50% (v/v) of complete Freund adjuvant was administered. Fourteen days later the same amount of protein was used mixed with incomplete Freund adjuvant. The third immunization was performed 14 days after the second with the same formulation as the second. Ten days after the last immunization, total blood was collected and incubated for 30 min at room temperature. The blood was centrifuged at 3,000 × *g* for 10 min and the sera was aliquoted in 50 μl volumes and stored at -20°C until their use. The antiserum was titrated and used in western blotting experiments.

### Culture Filtrate Proteins (CFP) and Cell Lysate Obtention

Mycobacteria cultures at logarithmic growth were harvested at 6,000 × *g* for 10 min. The supernatant and pellet were processed for CFP and cell lysate preparations, respectively. The supernatant was filtered through a 0.22 μm filter and then concentrated by centrifugation using a 10 kDa (Amicon) centricon filter at 7,000 × *g* for 30 min at 4°C. Glycerol was added to the obtained CFP to a final concentration of 20% and CFP was stored at -20°C until use. The culture pellet was resuspended in PBS buffer and sonicated in an ice bath twice for 1 min to obtain cell lysate. The cell lysate was adjusted to 20% glycerol and stored at -20°C.

### Western Blotting

After electrophoresis of the proteins by PAGE, under denaturing or non-denaturing conditions, the separated proteins were electrotransferred to a nitrocellulose membrane. The membrane was blocked with an incubation of 2 h with PBS containing 5% skimmed milk at room temperature. Then the membranes were incubated overnight at 4°C with the mouse serum against r0076 diluted 1:500 in PBS containing 2% skimmed milk. The membrane was then washed three times with PBS buffer and incubated with 4 μg of secondary anti-Mouse-F (ab’) 2-xx-biotin (Molecular Probes) for 2 h at 37°C. Then, horse anti-mouse antibody conjugated with avidin-peroxidase (Sigma-Aldrich) was added and incubated for 1 h. The reaction was developed by adding 0.05% diaminobenzidine (DAB, Roche) in 10 ml of H_2_O_2_. The image was acquired with the help of Gel documentation system (Bio-Rad) and analyzed with Quantity One 4.5.6 software (Bio-Rad).

### Circular Dichroism (CD) Spectroscopy

Circular dichroism spectrum was collected using a Jasco-815 spectropolarimeter equipped with a temperature control device. The r0076 concentration was 5 μM in 50 mM NaH_2_PO_4_ buffer adjusted at pH 8.0 containing 50 mM NaCl. All data were collected using 1 mm quartz cuvette. The spectrum was recorded over the wavelength range from 195 to 260 nm. A total of eight accumulations were averaged to form the CD spectrum, using a scanning speed of 100 nm/min, a spectral bandwidth of 1 nm, and a response time of 0.5 s. The buffer contribution was subtracted in each experiment. Thermal denaturation of r0076 at pH 8.0 was characterized by measuring the ellipticity changes at 222 nm induced by a temperature increase from 20 to 90°C. The fraction of denatured protein (α) was calculated from the relationship: α = (θ_n_ - θ_obs_)/(θ_n_ - θ_d_) and α + β = 1, in which θ_obs_ is the ellipticity obtained at a particular temperature, and θ_d_ and θ_n_ are the values of the ellipticity characteristic of the denatured and native states, respectively.

### Dynamic Light Scattering (DLS)

The size of r0076 was examined by means of the Nano-ZS dynamic light scattering system (Malvern Instruments Ltd., Malvern, United Kingdom). This system employs a λ = 633 nm laser and a fixed scattering angle of 173°. The r0076 solution (1 mg/ml), in buffer 50 mM NaH_2_PO_4_ buffer adjusted at pH 8.0 containing 50 mM NaCl, was centrifuged at 16,000 × *g* for 10 min at room temperature, and subsequently loaded into a quartz cuvette prior to measurement. The temperature was raised from 20 to 90°C and the sample was allowed to equilibrate for 2 min in each temperature prior to DLS measurements. The hydrodynamic radius (*R*_H_) was determined from a second-order cumulant fit to the intensity auto-correlation function (size distribution by volume). The determined *R*_H_ was converted to molecular mass (kDa) based on the assumption of a spherical particle and using the Zetasizer software.

### Iron Oxidation Assays

Oxidation reactions were performed according to Khare et al. (2011) using a fresh solution of 0.1 M of HEPES, pH 6.5 containing 125 μM of ammonium ferrous sulfate. The recombinant protein was added to the buffer containing ferrous sulfate for a final concentration of 0.25 μM, and the optical density was monitored at 310 nm for 18 min at 37°C. At this wavelength, the Fe^3+^ is detected, and consequently, the amount of oxidation can be monitored. To determine the amount of oxidized iron, additional replicate reactions were performed, but ferrozine iron reagent was also added to the reaction. Ferrozine makes a complex with free ferrous iron in solution, resulting in a violet color solution that can be detected at 570 nm. Ferrozine was added at 3-min intervals to individual wells and the 570 nm was recorded. A ferrous iron concentration curve was generated by adding ferrozine to different Fe^2+^ concentrations and recording the optical density (O.D.) at 570 nm. The experimental readings were converted to concentration based on the generated curve. The concentration at time zero was considered 100%, and the remaining concentrations were transformed in percentages relative to time zero. In all oxidation reactions, the 50 mM NaH_2_PO_4_ buffer adjusted to pH 8.0 containing 50 mM NaCl was used as negative control.

### Ethical Committee

The study was approved by the Ethics Committee for Animal use (CEUA: Comite de Ética no uso de animais; #229/11) of the Universidade Federal de Goiás (UFG), Goiânia, Brazil.

### Statistical Analysis

Comparison between means was assayed for variance (ANOVA) and non-paired *t*-test using Prism software version 6.0c (GraphPad). Values of *p* < 0.05 were considered statistically significant.

## Results

### Mycobacteria Belonging to the *Mycobacterium abscessus* Complex Have Two Genes Possibly Coding for Ferritin Proteins

**FIGURE 1:**
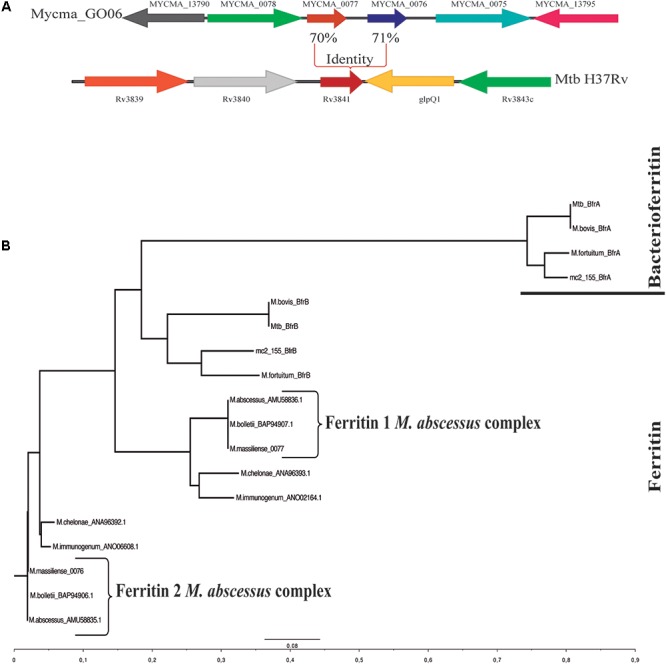
Genomic organization of the *Mycobacterium abscessus* subsp. *massiliense* genes *mycma_0076* and *mycma_0077* and phylogenetic tree obtained from alignments of ferritin and bacterioferritin protein sequences. **(A)** Genomic organization of the *M. abscessus* subsp. *massiliense* genes similar to *M. tuberculosis* H37Rv Rv3841. **(B)** Phylogenetic tree constructed from ferritin (BfrB) and bacterioferritin (BfrA) mycobacterial protein sequences performed on ClustalX. The protein sequences were obtained from NCBI BLAST searches for proteins with similarity to ferritin and bacterioferritin from *M. tuberculosis* H37Rv.

To identify possible genes coding for bacterioferritin and ferritin in the *M. abscessus* subsp. *massiliense* genome, a BLAST using the genes *bfrA* (Rv1876) and *bfrB* (Rv3841) from *M. tuberculosis* H37Rv performed against *M. abscessus* genomes and *M. abscessus* subsp. *massiliense* did not present any gene with significant similarity to the Rv1876 gene (**Supplementary Table S1**). However, *M. abscessus* subsp. *massiliense* has two genes with similarities higher than 70% to the Rv3841 gene (**Table 1**). Both *mycma_0076* and *mycma_0077* genes (**Figure 1A**) are located in tandem in the genome. Similar results were seen for other *M. abscessus* subspecies and other non-tuberculosis mycobacteria (NTM). A phylogenetic tree with the bacterioferritin and ferritin protein sequences from different mycobacteria species was constructed (**Figure 1B**). This shows that mycobacteria species closest to the group of *M. abscessus* may have two ferritins, while *M. tuberculosis* and other mycobacteria have one of both ferritin and bacterioferritin proteins. Thus, *M. abscessus* subsp. *massiliense* and other closely related genetic mycobacterial species do not have genes that are similar to the bacterioferritin gene *bfrA* (Rv1876) from *M. tuberculosis*, which suggests for the first time that mycobacteria from the *M. abscessus* complex and their closely related species have two genes possibly coding for ferritin.

**Table 1:** BLAST results from similarity search for *Mycobacterium tuberculosis* H37Rv Rv3841 gene.

Strain	Gene	Query cover	Identity	Location in genome
*M. abscessus* subsp. *massiliense* GO 06	*mycma_0076*	86%	71%	4557641 – 4558110
	*mycma_0077*	83%	70%	4556946 – 4557395
*M. abscessus* subsp. *abscessus*	A3O03_00650	86%	71%	129762 – 130307
	A3O03_00655	83%	70%	130479 – 131036
*M. abscessus* subsp. *bolletii*	MMASJCM_0130	86%	70%	127112 – 127581
	MMASJCM_0131	83%	69%	127827 – 128276
*M. chelonae*	BB28_00635	86%	70%	124938 – 125483
	BB28_00640	79%	70%	125655 – 126212
*M. immunogenum*	BAB75_00915	86%	69%	184826 – 185371
	BAB75_00920	89%	69%	185543 – 186100
*M. fortuitum*	XA26_58160	95%	76%	5966689 – 5967212
*M. smegmatis* mc^2^ 155	LJ00_31750	95%	76%	6492666 – 6493211
*M. bovis*	LH58_20775	100%	99%	4272259 – 4272804

*Alignments were performed with Basic Local Alignment Search Tool (BLAST) program at http://blast.ncbi.nlm.nih.gov, using the nucleotide BLAST algorithm.*

### Molecular Dynamics Evaluation of 0076 and 0077 Proteins Demonstrate a Ferritin Like Protein

To correlate the genes *mycma_0076* and *mycma_0077* with ferritin, their hypothetic structures were modeled and analyzed by molecular dynamics (MD). Initial models of 0076 and 0077 proteins were built from I-Tasser server using as principal templates (PDB files) 3qd8A (Crystal structure of *M. tuberculosis* BfrB), 1vlgA (Crystal structure of Ferritin from *Thermotoga maritima*), 3unoA (*M. tuberculosis* ferritin homolog, BfrB), and 1z6oA (Crystal Structure of *Trichoplusia ni* secreted ferritin). The information from each template compared to 0076 and 0077 proteins are shown in (**Table 2**). The best model (model 1) for 0076 and 0077 structures had a *C*-score of 1.13 and 0.92, respectively. These values provide an estimate for TM-score above 0.84 and an RMSD below 3.5 Å for both models. These predicted values indicate that the models determined by ITASSER have a great chance of representing the expected native structures for the 0076 and 0077 proteins.

**Table 2:** Accuracy of models and threading templates information.

Proteins Mycma	Accuracy of the model 1			Identity^∗^ (coverage^#^) from threading templates			
	*C*-score	TM-score (estimated)	RMSD (estimated)	3qd8A	1vlgA	3unoA	1z6oA
0076	1.13	0.87 ± 0.07	2.9 ± 2.1 Å	0.63 (0.95)	0.26 (0.91)	0.64 (0.96)	0.20 (0.93)
0077	0.92	0.84 ± 0.08	3.4 ± 2.3 Å	0.58 (0.93)	0.22 (0.89)	0.58 (0.93)	0.20 (0.92)

*^∗^Identity is the percentage sequence identity of the whole template chains with query sequence. ^#^ Coverage represents the coverage of the threading alignment and is equal to the number of aligned residues divided by the length of query protein.*

**FIGURE 2:**
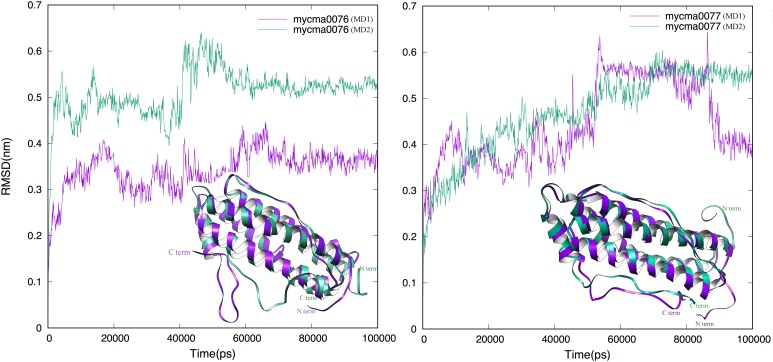
Root-mean-square deviation (RMSD) dynamic profiles obtained for the MD simulations of 0076 and 0077 protein structures over 100 ns. The MD1 (purple) and MD2 (green) are superimposed in the ribbon mode in insert figure.

The MD simulations from theses initial models were performed to achieve stability and/or improve the structure quality of them. In **Figure 2**, the RMSD evolution from initial models is shown for 0076 and 0077. For both proteins, after 60 ns, a transient stability could be verified for them. Just one simulation from 0077 protein had high fluctuations after 60 ns, which is mainly associated to moves from the residues located at the N and C-terminal (**Figure 2**).

**FIGURE 3:**
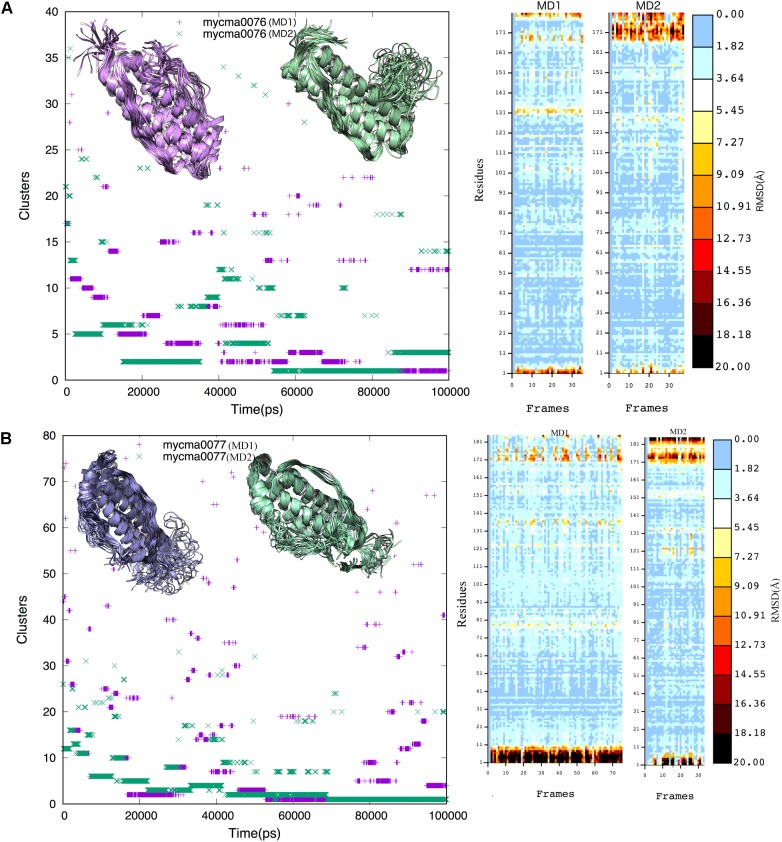
Cluster analysis of the 0076 **(A)** and 0077 **(B)** protein trajectories obtained over 100 ns. A cutoff of 0.2 nm was selected to include the main structures during the simulations. The cluster structures were determined using the non-hydrogen-atom RMSD values. Insert figures represent the cluster structures from MD1 (purple) and MD2 (green) independent simulations.

**FIGURE 4:**
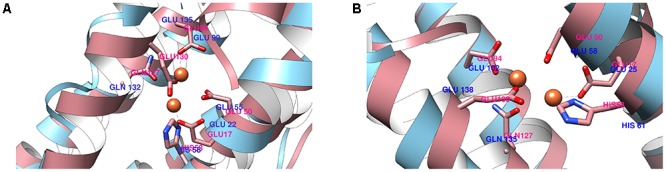
Structural alignment of the ferritin active site (pink) between 0076 **(A)** and 0077 **(B)** proteins against *Helicobacter pylori* ferritin.

From the trajectory of each simulation, cluster analysis of the conformations with a cutoff of 2 Å helped identify multiple conformations that could represent their flexibility. We selected only the center structure of the cluster most common during the simulations to represent each protein. **Figure 3** shows the clusters obtained for structures over time. For 0076 simulations (**Figure 3A**), cluster number one (most frequent structure) appeared only after about 60 ns, which remained stable until the end of the simulations. The same feature was observed for 0077 simulations (**Figure 3B**). In MD1 simulation of 0077 protein, the number of clusters observed between 60 ns and 80 ns fell from 70 to less than 20 (**Figure 3B**). Outside this interval, more intense structural fluctuations occur around N terminal region. The quality of the selected structures was measured by molprobity score, which indicates a better quality for the structure when its value tends to zero. **Table 3** shows that the quality of the models (model 1) had comparable molprobity scores from high-resolution structures. The highest value was 1.65 for 0077 (DM2) model, which is still a very common value in high resolution structures. The structural alignment of the likely active site of the *Helicobacter pylori* ferritin structure (PDB id 3bvi – chain C) and proteins 0076 and 0077 is shown in **Figure 4**.

**Table 3:** Molprobity score and ferritin active site key residues for proteins 0076 and 0077.

Proteins	Clashscore^$^	MolProbity score^∗^ (&)	% secondary structures			Key residues to ferritin active site
			Helix	Sheet	Others	
0076 (MD1)	0.0 (100th)	1.33 (98th)	65.20	0	34.8	GLU22, GLU55, HIS58, GLU99, GLN132, GLU135
0076 (MD2)	0.36 (99th)	1.11 (100th)	61.30	2.2	36.5	
0077 (MD1)	1.06 (99th)	1.46 (96th)	61.60	0	38.4	GLU25, GLU58, HIS61, GLU102, GLN135, GLU138
0077 (MD2)	2.47 (99th)	1.65 (91st)	58.40	0	41.6	
3bvi_C	1.43 (100th)	0.96 (100th)	71.70	0	28.3	GLU17, GLU50, HIS53, GLU94, GLN 127, GLU130

*^$^Clashscore measured from molprobity program. ^∗^Molprobity scores after molecular dynamics simulations. ^&^Percent scores observed in high resolution structures.*

### Evaluation of *mycma_0076* and *mycma_0077* Genes Expression

**FIGURE 5:**
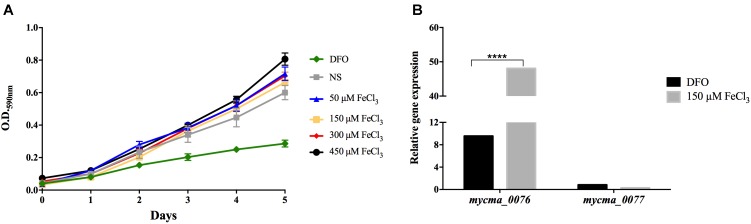
*Mycobacterium abscessus* subsp. *massiliense* growth **(A)** and relative gene expression of the genes *mycma_0076* and *mycma_0077* **(B)** in different iron concentrations. *M. abscessus* subsp. *massiliense* was grown in minimal media containing different iron concentrations at 37°C under agitation for 5 days. During this period, the growth was monitored by OD 590 nm readings. After incubation, total RNA was extracted and the expression of *mycma_0076* and *mycma_0077* was analyzed by quantitative RT-PCR with SYBR green under high (150 μM FeCl_3_) and low (DFO) iron conditions. The relative gene expression was determined by the Delta Delta Ct (2^-ΔΔCt^) method using the expression of the *16s* rRNA gene as normalizer and the growth under non-supplemented (NS) condition as calibrator. ^∗∗∗∗^*p* < 0.0001 indicate statistically significant difference between groups.

Bacteria require a mechanism for iron storage for efficient homeostasis of this ion and to avoid the deleterious effects of iron excess (Pandey and Rodriguez, 2012; Reddy et al., 2012). *M. abscessus* subsp. *massiliense* growth did not alter in different iron concentrations, ranging from minimal concentrations to excess conditions, such as 450 μM FeCl_3_ (**Figure 5A**). However, when iron was completely removed from the media, the mycobacteria growth was seriously compromised (**Figure 5A**). Thus, *M. abscessus* subsp. *massiliense* has mechanisms for iron homeostasis that allows this bacterium to grow in conditions of iron overload.

As *mycma_0076* and *mycma_0077* genes possibly correspond to ferritin genes, their expression was evaluated during *M. abscessus* subsp. *massiliense* growth in different iron concentrations. Surprisingly, the *mycma_0076* gene had its expression up regulated 50 times in high iron concentrations, while *mycma_0077* gene was not induced under those conditions (**Figure 5B**). Thus, only *mycma_0076* gene seemed to have a positive correlation between iron levels and expression (**Figure 5B**).

### The Expression of *mycma_0076* and *mycma_0077* Genes Is Modulated During Macrophage Infection

**FIGURE 6:**
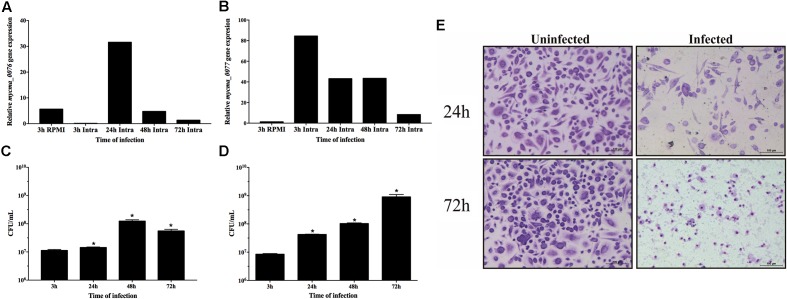
Relative gene expression of *mycma_0076* **(A)** and *mycma_0077* **(B)** genes and growth of *M. abscessus* subsp. *massiliense* during infection of BMDM. **(A,B)** Macrophages were infected at a multiplicity of infection of 1:10 with *M. abscessus* subsp. *massiliense*, and total RNA was extracted at 3, 24, 48, and 72 h after infection. Expression of *mycma_0076* and *mycma_0077* was analyzed by quantitative RT-PCR with SYBR green. The relative gene expression was determined by the delta delta Ct (2^-ΔΔCt^) method using the expression of the *16s* rRNA gene as normalizer and the bacilli obtained from supernatant of macrophage infection after 3 h as calibrator. **(C)** After 3, 24, 48, and 72 h of macrophage infection the macrophages were washed, lysed with water and plated on MH agar for CFU determination. **(D)** After 3, 24, 48, and 72 h of macrophage infection, the supernatant was collected for CFU quantification of extracellular bacilli. ^∗^*p*-value < 0.05 indicate statistically significant difference between groups as compared with 3 h post infection. **(E)** After 24 and 72 h of incubation, uninfected and infected cells were stained and analyzed by light microscopy (Leica Application Suite v.4.4.0) to observe cell damage.

In order to understand if the differential expression observed *in vitro* was also used by *M. abscessus* subsp. *massiliense* to overcome the infection, the expression of *mycma_0076* and *mycma_0077* genes were evaluated during macrophage infection. In contrast to the *in vitro* observations, expression of both genes was induced during macrophage infection, but these genes were differently modulated (**Figures 6A,B**). While the expression of *mycma_0076* gene was reduced 3 h after macrophage infection (**Figure 6A**), the *mycma_0077* had its expression up regulated 80 times (**Figure 6B**). At 24 h of infection both genes had similar levels of expression, however, after 48 h the expression of gene *mycma_0076* was reduced again, while the expression of *mycma_0077* remained highly expressed (**Figures 6A,B**). After 72 h of infection, both genes were expressed at lower levels compared to 48 h (**Figures 6A,B**). These results suggested that the expression of *mycma_0076* and *mycma_0077* genes could be related to the establishment of infection, but their possible role in this process is not redundant. Moreover, it was observed that expression of *mycma_0076* and *mycma_0077* increased accompanied by the growth of bacilli inside of macrophages (24 h) (**Figures 6A–C**). However, the bacterial number within macrophage culture reduced after 72 h (**Figure 6C**), as did the expression both genes were (**Figures 6A,B**). It is of important notice that the observed decrease in intracellular bacteria was accompanied by increase in extracellular bacilli (**Figure 6D**) and macrophage death (data not shown). These data suggest that the expression of both genes are important for bacilli growth inside of macrophages, differently to extracellular growth as in RPMI when the *mycma_0076* gene was predominantly expressed (**Figures 6A,B**).

### Protein 0076 Cytolocalization in *M. abscessus* subsp. *massiliense*

**FIGURE 7:**
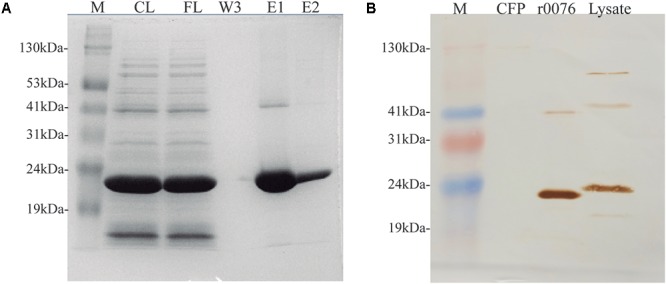
*Mycobacterium abscessus* subsp. *massiliense* recombinant protein r0076 expression, purification, and localization. **(A)** SDS-PAGE analysis of different purification fractions of r0076. M, prism protein marker (Amresco); CL, cell lysate; FL, flow-through fraction; W, third wash fraction; E1 and E2, elution fractions. **(B)** *M. abscessus* subsp. *massiliense* grown for 5 days in MH at 37°C was harvested and its supernatant culture filtrate (CFP) and cell pellet (Lysate) where analyzed on a western blotting using polyclonal antibodies raised against r0076. The purified recombinant protein was used as control (r0076). M, prism protein marker (Amresco); CFP, culture filtrate protein.

Both *mycma_0076* and *mycma_0077* genes from *M. abscessus* subsp. *massiliense* were separately cloned in pET28a(+) plasmid, expressed in *E. coli* and the recombinant proteins were purified (**Figures 7A** and **Supplementary Figure S2**). While recombinant 0076 (r0076) protein was easily obtained in its soluble form, r0077 had very low solubility and yield. Consequently, some experiments for ferritin characterization was performed only for r0076. The protein 0076 was detected by specific polyclonal antibodies only in the cellular fraction of *M. abscessus* subsp. *massiliense* (**Figure 7B**).

### Recombinant 0076 Protein Complex Formation

**FIGURE 8:**
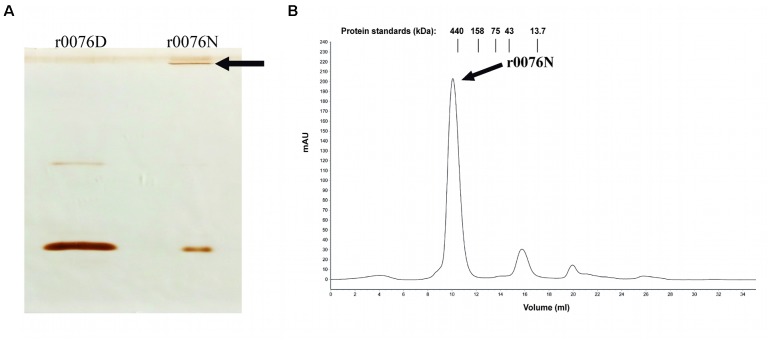
Recombinant protein r0076 forms a high molecular mass complex. **(A)** Western blotting of non-denaturing gel using polyclonal antibodies against r0076. The r0076 protein was boiled in denaturing buffer (r0076D) or only resuspended in non-denaturing buffer (r0076N). **(B)** Gel filtration chromatographic profile of native r0076 elution on a Superdex 200 10/300 GL column.

The results presented above support the function of the protein 0076 as a ferritin. Several studies have shown that ferritin proteins require the formation of an oligomeric structure to perform iron oxidation and storage (Levi et al., 1988; Khare et al., 2011, 2013). We could show that this was the case for r0076 by performing western blotting of the protein under native polyacrylamide gel conditions. As shown in **Figure 8A**, r0076 forms a high molecular mass protein. Upon gel filtration analysis, r0076 elutes as single peak of apparent molecular mass of 480 kDa (**Figure 8B**) similar to ferritins.

### Recombinant Ferritin From *M. abscessus* subsp. *massiliense* (r0076) Forms Stable Oligomers in Solution

**FIGURE 9:**
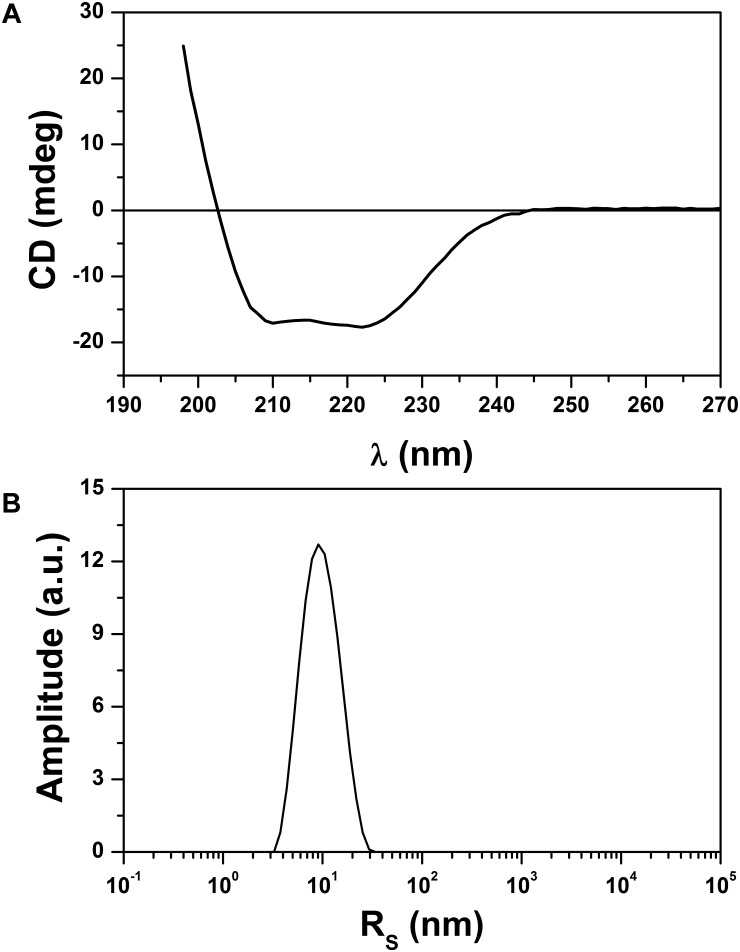
Analysis of secondary and quaternary structures. **(A)** Circular dichroism (CD) spectrum of the recombinant 0076 at pH 8. **(B)** Size distribution by intensity for recombinant 0076 where DLS runs were conducted at pH 8.

Circular dichroism spectroscopy was used to analyze the secondary structure of the r0076 in solution at pH 8.0 (**Figure 9A**). The CD spectrum of r0076 is characterized by two minima at 210 ± 1 nm and 222 ± 1 nm, a maximum near 200 ± 1 nm, and a negative to positive crossover at 201 ± 1 nm. The negative minimum was around 210 and 222 nm, which strongly indicate the presence of α helices, comparable to the secondary structure of other ferritins (Khare et al., 2011, 2013). As a next step, the quaternary structure of r0076 was analyzed in solution at pH 8.0 by dynamic light scattering (DLS). When r0076 was analyzed by DLS the observed profile was characteristic of a monodisperse solution (**Figure 9B**). The value of hydrodynamic radius (*R*_H_) determined for r0076 was 8.0 ± 0.5 nm, certainly corresponding to an oligomeric form of the protein in solution. This result is consistent with the size-exclusion chromatography profile obtained for r0076 (**Figure 8B**).

### Influence of Temperature on r0076 Stability and Compactness

**FIGURE 10:**
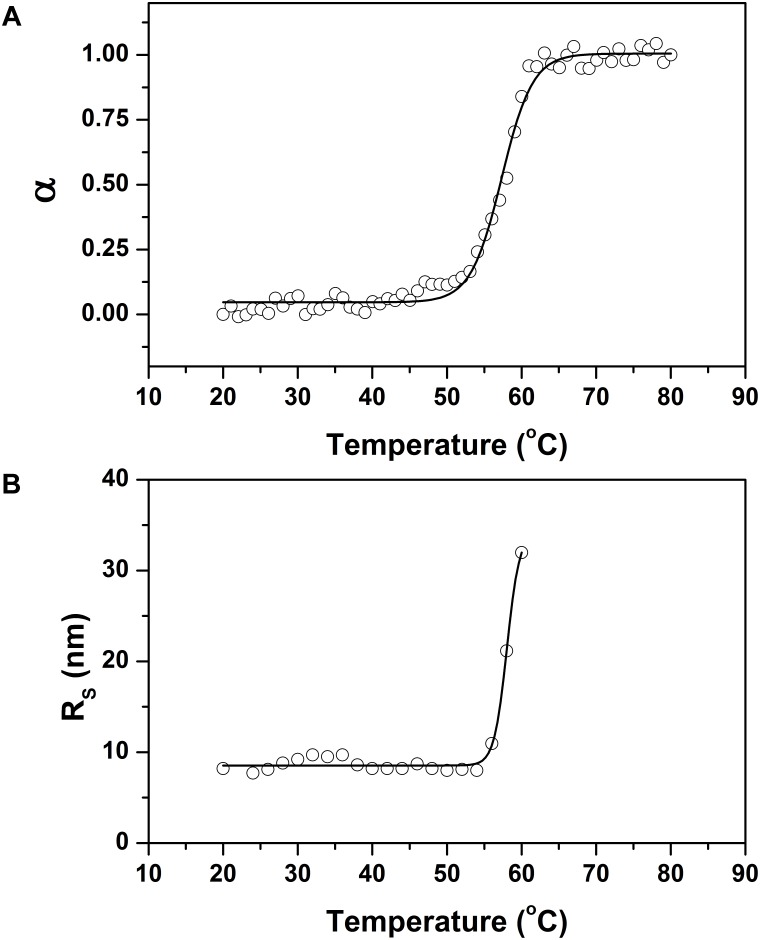
Thermal stability of recombinant 0076. **(A)** Thermal denaturation of recombinant 0076 at pH 8 was monitored by measuring the ellipticity at 222 nm as a function of temperature. **(B)** The hydrodynamic radius (*R*_H_) of r0076 at pH 8 as a function of temperature.

The thermostability of the r0076 at pH 8.0 was monitored following changes in the ellipticity at 222 nm (**Figure 10A**). The spectrum remained constant at temperatures below 55°C. However, the spectrum was progressively altered when the temperature was increased above 55°C, which indicates loss of the regular secondary structure. The melting temperature (*T*_m_), value determined by CD spectroscopy for r0076, was 57 ± 1°C (**Figure 10A**). The structural alteration observed by CD spectroscopy was accompanied by DLS analyses. **Figure 10B** shows the variation of *R*_H_ as a function of temperature for r0076 at pH 8.0. The *R*_H_ of r0076 exhibited minimal temperature dependence between the ranges of 20 to 55°C. However, when r0076 was incubated at temperature values above 55°C, the *R*_H_ increased significantly, suggesting the formation of aggregates as a consequence of the denaturation process. The thermal denaturation process was essentially irreversible in the conditions described in this study (data not shown).

### Recombinant r0076 and r0077 Proteins Promote Oxidation of Fe^2+^ Into Fe^3+^

**FIGURE 11:**
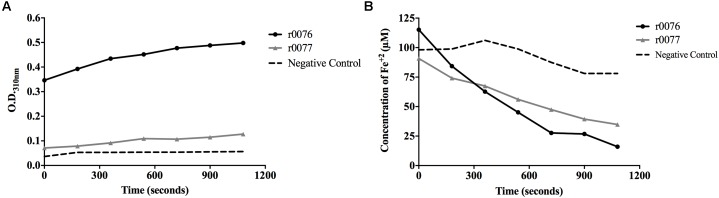
Recombinant proteins r0076 and r0077 have iron oxidizing activity. **(A)** The oxidation of Fe^2+^ into Fe^3+^ was monitored by the increase in absorbance at 310 nm. **(B)** Oxidation of Fe^2+^ into Fe^3+^ rates through the decrease of absorbance by the ferrozin-Fe^2+^ complex at 570 nm wavelength. The negative control was buffer 50 mM NaH_2_PO_4_ buffer adjusted at pH 8.0 containing 50 mM NaCl.

The capacity of the recombinant proteins to promote the oxidation of ferrous iron was evaluated by incubating them with Fe^2+^ and observing the increase in optical density at 310 nm. Both r0076 and r0077 proteins were capable to oxidize ferrous iron (**Figure 11A**), but the activity of r0076 was much greater than r0077. The r0076 protein oxidized 25% of the available Fe^2+^ (**Figure 11B**) after 3 min, while r0077 protein oxidized only 16% during the same time (**Figure 11B**). After 18 min, the r0076 protein oxidized more than 80% of the available Fe^2+^ while, r0077 oxidized 55%. These results demonstrate that both r0076 and r0077 proteins are capable of oxidizing Fe^2+^ into Fe^3+^, evidencing their activities as ferritins.

## Discussion

In the last decades, the *M. abscessus* complex has emerged as a human pathogen (Petrini, 2006; Medjahed et al., 2010). Its ability to infect and persist inside phagocytic cells and in the extracellular environment indicates that this bacterium has evolved to adapt and establish infection in humans (Bernut et al., 2014; Helguera-Repetto et al., 2014; Brambilla et al., 2016). In this study, we showed that *M. abscessus* subsp. *massiliense* has two ferritins similar to the Mtb ferritin that might be important for intracellular iron homeostasis, which in turn is crucial for successful infection.

It has been shown that *E. coli* and *Haemophilus influenza* have more than one gene coding for ferritin, while *M. tuberculosis* and *M. smegmatis* have only one (Andrews, 1998; Bou-Abdallah et al., 2014). Here, we showed for the first time that bacteria from the *M. abscessus* complex have two genes coding for ferritins and none for bacterioferritin (**Figure 1**, **Table 1**, and **Supplementary Table S1**). It was observed that the proteins-coded by *mycma_0076* and *mycma_0077* genes do not have the methionine (Met) residue at position 52 as observed in bacterioferritin from *M. tuberculosis* (data not shown). The absence of Met-52 may render these proteins unable to bind heme (Gupta et al., 2009; Yasmin et al., 2011) as shown in Met-52 *M. tuberculosis* mutants (Gupta et al., 2009; Khare et al., 2017). Absence of the gene with similarity to Rv1867 gene (**Supplementary Table S1**) and lack of Met-52 raise the possibility that *M. abscessus* subsp. *massiliense* do not have bacterioferritin homolog.

To confirm if the proteins 0076 and 0077 might support ferritin functions, structural and molecular dynamic analyzes were performed. Considering model-1 structures from MD1 and MD2 simulations, the RMSD (CA atoms) between them is 7.03 Å and 6.24 Å for 0076 and 0077 proteins, respectively. The 0076 and 0077 proteins have about 60% of α helix secondary structures and low content of β-sheet secondary structures (see **Table 2**). When residues involved in segments other than the helices are removed from the RMSD estimates, RMSD values fall to 1.20 Å (113 CA atoms) and 1.23 Å (111 CA atoms). This becomes clear in the RMSD fluctuations for residues shown in **Figures 2**, **3** from all clusters. RMSD above 5.0 Å occurred mainly in the segments involved in the N and C terminus, except around residues 130–135, where sensitive structural fluctuations were observed. This may have provided instability in this region and even partial loss of the helix structure, such as illustrated in **Supplementary Figure S1**. The slight fluctuation of the residues involved in segment 130–135 may be due to the templates used to construct the model (3d8A) whose irons were present in the structure and not included in the simulations. On the other hand, it may also be associated with the flexibility expected to assist in the conformational rearrangement of this region to accommodate iron ions and make them more stable. Above all, irrespective of the presence of iron, the conformations of the proteins were stable at their sites, as expected for the positions of the key residues of a ferritin (**Figure 4**). This reinforces the idea that these structures are not dependent on iron for their stability and formation (Stookey, 1970; Waldo and Theil, 1993; Theil et al., 2000).

Although the main crystal structure used to construct the models was not solved with the presence of iron in this region (3qd8A), the MD simulations showed the importance of these ions for the stability of this site. **Table 2** shows that the main residues from *Helicobacter pylori* ferritin structure (GLU17, HIS53, GLU50 GLU94, GLN 127, GLU 130) are conserved in 0076 and 0077 proteins (Cho et al., 2009). Additionally, we observed that the residues involved in the self-assembly and stability are the same as those recently reported by Khare et al. (2013). The 3D position of theses residues for both structures are highly correlated with that observed for *Helicobacter pylori* ferritin structure (**Figure 4**), which strongly supports the ferritin activity and the same iron binding mechanism of these two proteins.

The expression of the *mycma_0076* and *mycma_0077* genes, evaluated *in vitro* with different iron concentrations was found to be differently regulated, suggesting different roles in iron homeostasis for this *Mycobacterium*. Furthermore, *M. abscessus* subsp. *massiliense* was able to grow in highly toxic iron concentrations (450 μM FeCl_3_), which indicates the presence of a homeostasis mechanism (**Figure 5A**). A recent transcriptomic analysis study of *M. abscessus* subsp. *abscessus* grown in the presence of cystic fibrosis patient sputum listed the different expression of the genes similar to *mycma_0076 (MAB_0126c)* and *mycma_0077 (MAB_0127c*), although that study did not investigate ferritins specifically (Miranda-CasoLuengo et al., 2016). In this previous study, the gene *MAB_0126c* was induced when grown with patient sputum as the stress condition. We found that the gene *mycma_0076* was also induced under *in vitro* conditions with high stressing concentration of iron (**Figure 5B**). Similarly, the requirement of ferritin expression to reduce the effects of oxidative damage was observed in Mtb (Pandey and Rodriguez, 2012, 2014). Thus, among other functions, ferritins play an important role in the resistances against stress conditions (Khare et al., 2017).

The different expression profiles of both *mycma_0076* and *mycma_0077* genes under different iron concentrations (**Figure 5B**), suggests that the proteins coded by theses genes have different roles in iron homeostasis. Recent studies have shown that Mtb ferritin and bacterioferritin have different roles in the cellular homeostasis, suggesting that the presence of two classes of ferritins is non-redundant and important for virulence (Khare et al., 2017). The interesting question is why does *M. abscessus* subsp. *massiliense* have two similar proteins of the same ferritin group? The overexpression of 0076 in high iron concentrations suggests that this protein may be involved in the storage of the ion providing protection to the bacilli from iron-mediated toxicity (**Figure 5B**).

Nonetheless, both *mycma_0076* and *mycma_0077* genes were expressed during macrophage infection, but they were differentially regulated according to the time of infection, which indicates that inside of macrophages, *M. abscessus* subsp. *massiliense* find a different microenvironment as compared with the medium, requiring different expression of those genes. It was observed during macrophage infection that the *mycma_0077* gene was expressed at higher levels, when compared with the levels of expression the *mycma_0076* (**Figures 6A,B**). That difference suggested that the expressions of *mycma_0076* and *mycma_0077* genes and their respective involvement in iron homeostasis are largely dictated by the microenvironment surrounding the cell, and they may play different or redundant but independent roles in iron management. Additionally, during macrophage infection, the *M. abscessus* find a more oxidizing environment compared to an uninfected cell, but the bacilli grow better in this condition (Oberley-Deegan et al., 2010). Moreover, it was demonstrated that an enhanced oxidative stress happens at 24 h post infection of macrophages when the bacilli growth increase (Oberley-Deegan et al., 2010). Our results demonstrated that the *mycma_0076* and *mycma_0077* genes were induced at the same time during macrophage infection (**Figure 6**). Furthermore, we have previously shown that the infection of *M. abscessus* subsp. *massiliense* induced high levels production of NO by spleen and liver cells (Martins de Sousa et al., 2010). Our findings raise the possibility that induction of the *mycma_0076* and *mycma_0077* genes expression during macrophage infection could be related to the resistance of these mycobacteria from oxidative stress caused by the macrophage. Besides, it was demonstrated in *M. tuberculosis* that the ferritin provide protection against oxidative stress and the deletion of this protein increased the sensibility to oxidative damage (Pandey and Rodriguez, 2012; Khare et al., 2017).

After 72 h post infection the burden of bacilli inside macrophages reduced significantly as compared with 48 h (**Figure 6C**) and in the same time of infection, the expression of the *mycma_0076* and *mycma_0077* genes also were reduced (**Figures 6A,B**). An initial interpretation of this observation could be related to the control of infection by macrophages, however, it was observed that the mycobacteria was extravasating to the extracellular milieu (**Figure 6D**). It has been reported that the *M. abscessus* can induce apoptosis of macrophages as a mode of mycobacterial escape for release and growth at the extracellular milieu (Sohn et al., 2009; Bernut et al., 2014; Brambilla et al., 2016; Whang et al., 2017). To confirm that this was the case, infected and uninfected cells were stained and analyzed 24 and 72 h post incubation. Damaged macrophages were observed after 24 h of *M. abscessus* subsp. *massiliense* infection and increased as the infection progressed (**Figure 6E**) concomitant to the increase of extracellular bacteria (**Figure 6D**). This result indicates that *M. abscessus* subsp. *massiliense* can induce damage on macrophages and consequently be released to the extracellular environment when the expression of the *mycma_0076* and *mycma_0077* genes would not be as much necessary as within the intracellular environment.

To confirm the iron storage characteristics of the protein encoded by the *mycma_0076* and *mycma_0077* genes, the recombinant proteins were expressed in *E. coli*. While r0076 was obtained in the soluble form, r0077 remained mostly in insoluble form despite different attempts to obtain it in a soluble form. The CD spectrum obtained for r0076 is typical of proteins with high content of α-helical secondary structure (**Figure 9A**). Moreover, the formation of oligomers in solution (**Figure 9B**), together with the presence of bound iron ions, indicates that r0076 folded correctly. As seen in **Figure 8**, r0076 eluted as a major peak (**Figure 8B**) with an apparent molecular mass of 480 kDa. This result is broadly consistent with an oligomer composed of 24 subunits, whose theoretical expected mass for each subunit is 20 kDa. When r0076 was analyzed by DLS, a *R*_H_ of 8.0 ± 0.5 was determined. Assuming a spherical particle, the value determined for *R*_H_ correspond to a molecular mass of 437 ± 63 kDa, which would also be consistent with an oligomer composed of 24 subunits. The quaternary structures of several ferritins were found to be strikingly similar (Khare et al., 2011, 2013). The ferritin from *M. tuberculosis* exhibits a quaternary structure where 24 subunits assemble in octahedral 432-symmetric arrangements to form a roughly spherical protein shell (Khare et al., 2011).

The CD spectrum of r0076 was constant below 55°C; however, above this temperature there was a progressive loss of regular secondary structure (**Figure 10A**). Concomitantly, the *R*_H_ of r0076 exhibited minimal temperature dependence in the range of 20–55°C, while the *R*_H_ increased significantly when the protein was incubated at temperatures above 55°C (**Figure 10B**). Taken together, these results indicate that the increase in temperature does not induce r0076 dissociation prior to denaturation.

The main function of ferritin is to detoxify and store free cellular iron, which is accomplished by oxidation reaction at the ferroxidase center (Andrews, 1998). Molecular dynamics of both models obtained from the amino acid sequences of 0076 and 0077 proteins found conserved amino acid residues involved in the ferroxidase active site as shown for *H. pylori* crystallographic resolved ferritin protein (**Figure 4**) (Cho et al., 2009). We showed here that both proteins r0076 and r0077 are capable of oxidizing Fe^2+^ into Fe^3+^, confirming their ferritin function similarly to Mtb ferritin (**Figure 11**) (Khare et al., 2011, 2013).

Although the essentiality of the ferritin genes reported here can only be demonstrated by silencing their expression (knockdown them out for example), we have clearly shown for the first time that *mycma_0076* and *mycma_0077* genes codes for a ferritin.

## Conclusion

The genes *mycma_0076* and *mycma_0077* from *M. abscessus* subsp. *massiliense* code for bacterial ferritins, homologous to Mtb ferritin gene (Rv3841), that are differently modulated by iron concentration both *in vitro* and *in vivo.* Additionally, both proteins r0076 and r0077 were capable to oxidize Fe^2+^ into Fe^3+^ supporting an active ferroxidase center. The implications that *M. abscessus* complex has only ferritins and no bacterioferritins should be further explored.

## Author Contributions

FO, ADC, and VP carried out most of the experiments. RS performed the MD experiments and wrote the pertinent data of these experiments. WG and JA performed the CD and DLS experiments and wrote the results and discussion regarding this data. AJ-K and AK designed the experiments and supervised all work. FO, AJ-K, and AK wrote the manuscript. All authors revised the manuscript and approved the final version.

## Conflict of Interest Statement

The authors declare that the research was conducted in the absence of any commercial or financial relationships that could be construed as a potential conflict of interest.

## Footnotes

1 http://www.ncbi.nlm.nih.gov/genome/

2 http://blast.ncbi.nlm.nih.gov/Blast.cgi

3 http://www.ncbi.nlm.nih.gov/tools/primer-blast

## Supplementary Material

FIGURE S1 Secondary fluctuations structures over 100 ns from 0076 to 0077 proteins. **(A)** Secondary structures of 0076 MD1. **(B)** Secondary structures of 0076 MD2. **(C)** Secondary structures of 0077 MD1. **(D)** Secondary structures of 0077 MD2.

FIGURE S2 Solubilization of r0077 protein from inclusion bodies using sarkosyl and purification. **(A)** SDS-PAGE gel showing lysis of bacterial cells induced or not with 1 mM IPTG for 4 h. **(B,C)** SDS-PAGE gel showing solubilization of r0077 protein with various concentration of sarkosyl. **(D)** Purification of r0077 using His tag from soluble fraction obtained with 2.5% sarkosyl. **(E)** SDS-PAGE gel showing r0077 protein purified concentrated.

TABLE S1 BLAST results from similarity search for the Rv1876 gene from *M. tuberculosis* H37Rv.

TABLE S2 Primer sequences used to evaluate the expression of *M. abscessus* subsp. *massiliense* genes.
