# Supplementary figures and images for: Mycobacterium abscessus subsp. massiliense mycma_0076 and mycma_0077 Genes Code for Ferritins That Are Modulated by Iron Concentration
Source: Front Microbiol. 2018 Jun 1;9:1072. doi: 10.3389/fmicb.2018.01072 (PMC5992710; doi:10.3389/fmicb.2018.01072)

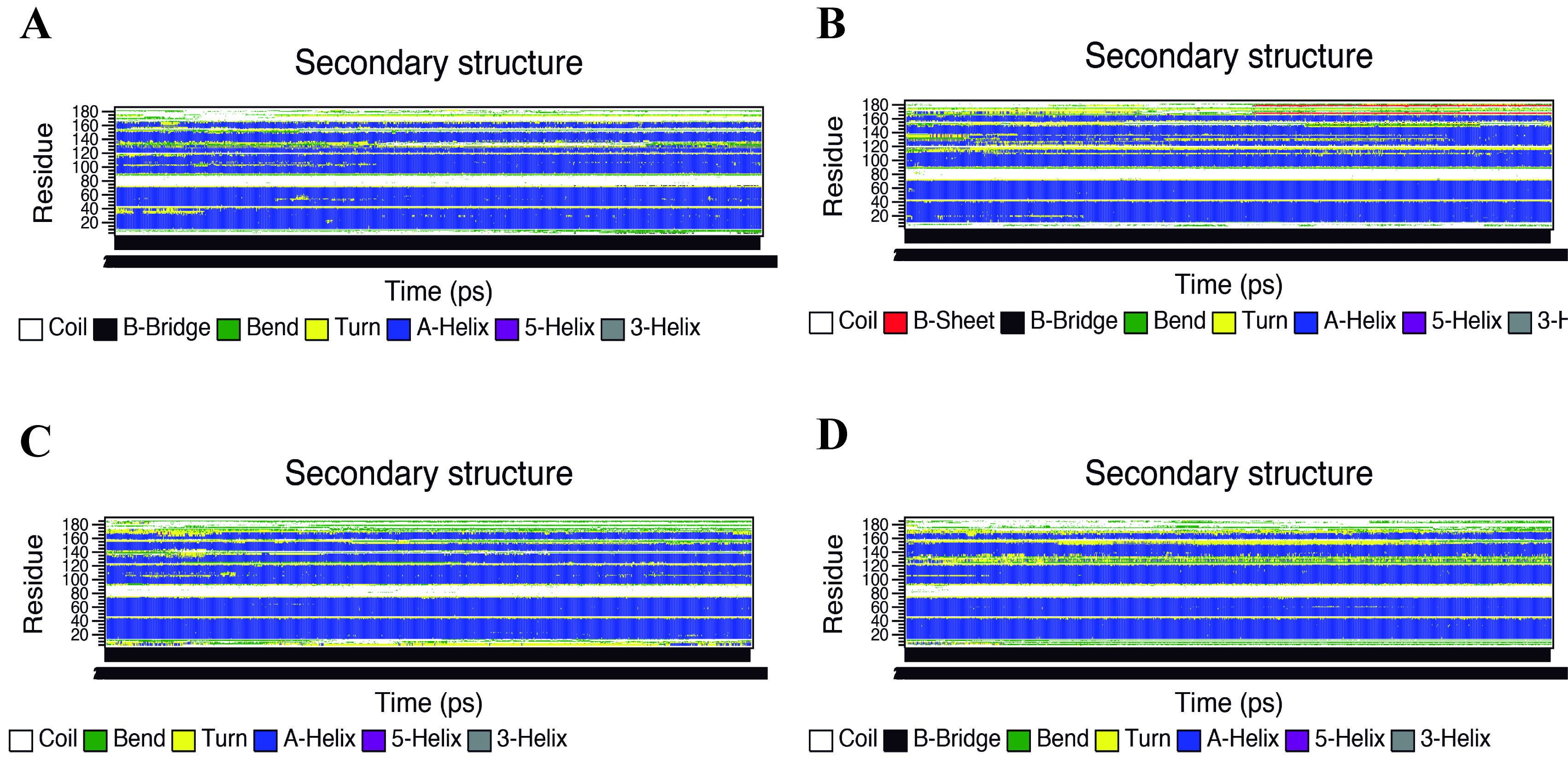

Supplement: FIGURE S1 — Secondary fluctuations structures over 100 ns from 0076 to 0077 proteins. (A) Secondary structures of 0076 MD1. (B) Secondary structures of 0076 MD2. (C) Secondary structures of 0077 MD1. (D) Secondary structures of 0077 MD2. [file Image_1.TIF]

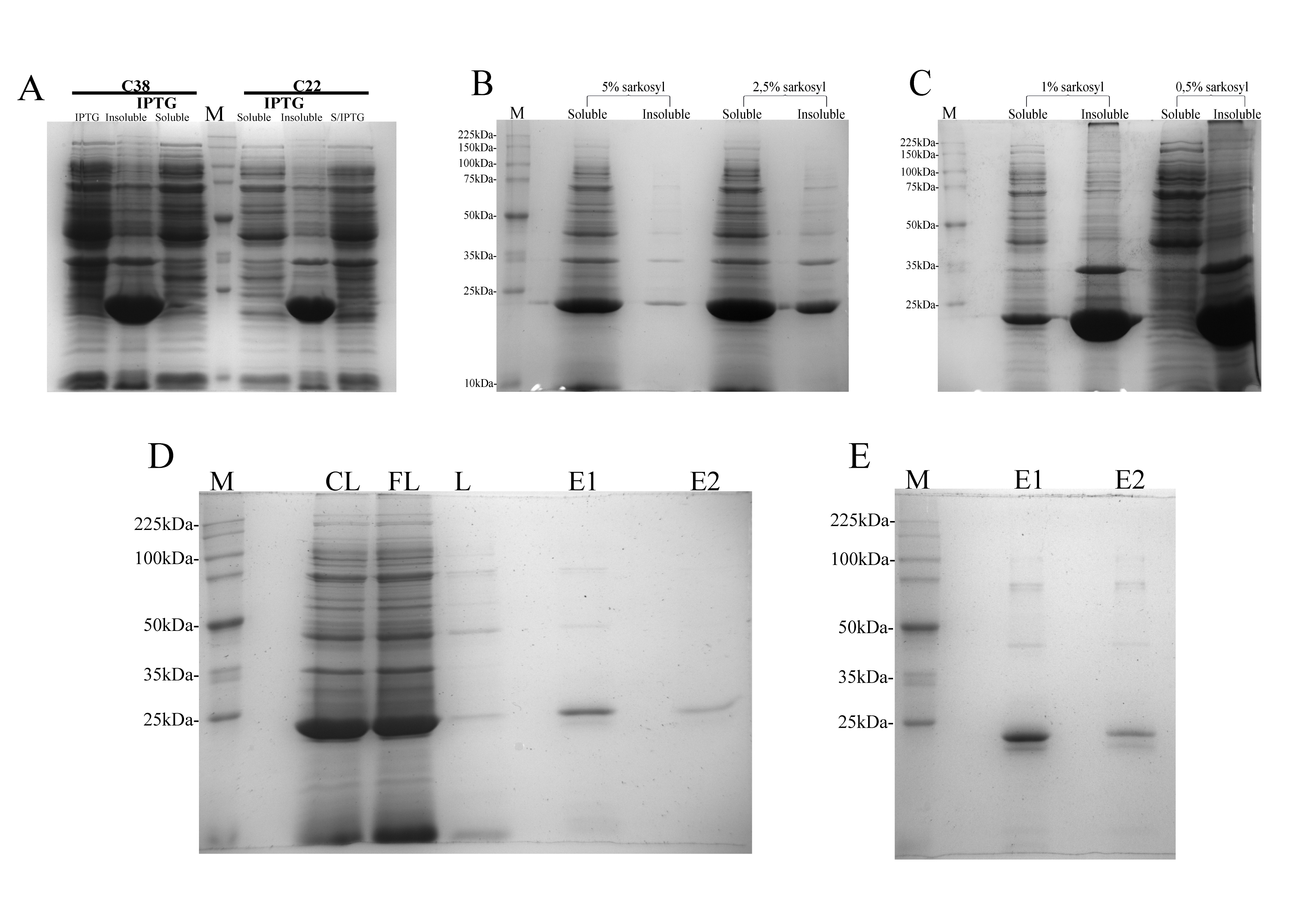

Supplement: FIGURE S2 — Solubilization of r0077 protein from inclusion bodies using sarkosyl and purification. (A) SDS-PAGE gel showing lysis of bacterial cells induced or not with 1 mM IPTG for 4 h. (B,C) SDS-PAGE gel showing solubilization of r0077 protein with various concentration of sarkosyl. (D) Purification of r0077 using His tag from soluble fraction obtained with 2.5% sarkosyl. (E) SDS-PAGE gel showing r0077 protein purified concentrated. [file Image_2.TIF]
