# Supplementary material for: Mycobacterium abscessus subsp. massiliense mycma_0076 and mycma_0077 Genes Code for Ferritins That Are Modulated by Iron Concentration
Source: Front Microbiol. 2018 Jun 1;9:1072. doi: 10.3389/fmicb.2018.01072 (PMC5992710; doi:10.3389/fmicb.2018.01072)
Supplement: TABLE S1 — BLAST results from similarity search for the Rv1876 gene from M. tuberculosis H37Rv. [file Data_Sheet_1.docx]

**Supplementary figures and table**

**Supplementary Table 1:** BLAST results from similarity search for the Rv1876 gene from *M. tuberculosis* H37Rv.

| Strain | Gene | Query cover | Identity | Location in genome |
| --- | --- | --- | --- | --- |
| *M. abscessus* subsp. *massiliense* GO 06 | None | None | None | None |
| *M. abscessus* subsp. *abscessus* | None | None | None | None |
| *M. abscessus* subsp. *bolletii* | None | None | None | None |
| *M. chelonae* | None | None | None | None |
| *M. immunogenum* | None | None | None | None |
| *M. fortuitum* | XA26_33300 | 98% | 78% | 3388202 – 3388681 |
| *M. smegmatis* mc^2^ 155 | LJ00_17740 | 99% | 77% | 6492666 – 6493211 |
| *M. bovis* | LH58_10055 | 100% | 100% | 2085052 - 2085531 |

**Alignments were performed with Basic Local Alignment Search Tool (BLAST) program at** [**http://blast.ncbi.nlm.nih.gov**](http://blast.ncbi.nlm.nih.gov)**, using the nucleotide BLAST algorithm.**

**Supplementary Table 2.** Primer sequences used to evaluate the expression of *M. abscessus* subsp. *massiliense* genes*.*

| Name | Sequence 5’- 3’ | | | Product size |
| --- | --- | --- | --- | --- |
| mycma_0076_Fwd | | GTTACCGAGCAGGTCACACA | 117pb | |
| mycma_0076_Rev | | GTCCATGAGCGCGACTTCTT |  |  |
| mycma_0077_Fwd | | AACGTAGCCATCACGATCCC | 102pb | |
| mycma_0077_Rev | | GGTGACGCGCTTTTCTTGTT |  |  |
| mycma_16s_Fwd | | AGCTCGTAGGTGGTTTGTCG | 214pb | |
